# The rediscovery of *Passiflora kwangtungensis* Merr. (subgenus *Decaloba* supersection *Disemma*): a critically endangered Chinese endemic

**DOI:** 10.3897/phytokeys.23.3497

**Published:** 2013-06-12

**Authors:** Shawn E. Krosnick, Xun-Lin Yu, Yunfei Deng

**Affiliations:** 1 Department of Biology, Southern Arkansas University, 100 East University Street, Magnolia, AR, 71753, U.S.A.; 2 Forestry Institute, Central South University of Forestry and Technology, Changsha, 410004, Hunan, China; 3 South China Botanical Garden, Chinese Academy of Sciences, No. 723, Xingke Lu, Tianhe Qu, 510650, Guangzhou, China

**Keywords:** China, *Decaloba*, *Disemma*, *Passiflora*, *Passiflora kwangtungensis*, Passifloraceae

## Abstract

*Passiflora kwangtungensis* is a critically endangered Chinese species known from Guangxi, Guangdong, and Jiangxi Provinces. The species belongs to *Passiflora* subgenus *Decaloba*, supersection *Disemma*, section *Octandranthus*. Field observations decreased rapidly during the 1970s to 1980s, and it was suspected that this species might have been extirpated due to repeated deforestation events throughout southern China. In recent years, however, small isolated populations of this species have been rediscovered in Hunan Province, representing new locality records for *Passiflora kwangtungensis*. New herbarium collections, color photographs, and silica gel collections have provided an unexpected opportunity to examine the evolutionary significance of this species. The current study presents a revised morphological description of *Passiflora kwangtungensis* based on fresh material, along with an updated distribution map. Using nrITS sequence data, preliminary insights into the phylogenetic position of *Passiflora kwangtungensis* are presented. Molecular data support the placement of *Passiflora kwangtungensis* within supersection *Disemma* section *Octandranthus*. However, the exact placement of *Passiflora kwangtungensis* within this lineage is unclear. The nrITS data suggest that *Passiflora kwangtungensis* may be sister to a clade containing *Passiflora* from China, Nepal, India, and Southeast Asia. Morphologically, *Passiflora kwangtungensis* displays the most similarity *Passiflora geminiflora* (Nepal, India) and *Passiflora henryi* (China). Lastly, conservation status and recommendations are made for *Passiflora kwangtungensis* following the IUCN Red List Criteria, where this species is classified as CR C1+C2a(i); D.

## Introduction

The genus *Passiflora* L. consists of ca. 526 species (Feuillet and MacDougal 2003) with native ranges throughout the southern United States, Mexico, Central, and South America. In addition, there are 24 species of *Passiflora* endemic to the Old World. The Old World species are recognized within two subgenera: subgenus *Tetrapathea* (DC.) Rchb. (Krosnick et al. 2009) and subgenus *Decaloba* (DC.) Rchb. Subgenus *Tetrapathea* consists of three species restricted to Australia, New Zealand, and Papua New Guinea. Subgenus *Decaloba* supersection *Disemma* (Labill.) J.M. MacDougal & Feuillet contains the remaining 21 species found throughout Asia, Southeast Asia, and the Austral Pacific (Krosnick and Freudenstein 2005). Supersection *Disemma* contains three sections: section *Octandranthus* Harms, with 17 Asian and Southeast Asian species, section *Disemma* (Labill.) J.M. MacDougal & Feuillet, with three Australian endemics, and lastly, the monotypic section *Hollrungiella* Harmsfrom Papua New Guinea. The largest section, *Octandranthus*, has its center of diversity in China with 13 of the 17 species in this clade distributed there. These species arefound in Yunnan, Guangdong, Guangxi, Jiangxi, Hunan, and Hainan Provinces (Wang et al. 2007). The native *Passiflora* in China exhibit high levels of endemism, seldom display overlapping distributions, and are in general extremely rare. Of those 13 species found in China, *Passiflora wilsonii* Hemsl., *Passiflora eberhardtii* Gagnep., *Passiflora jugorum* W.W. Sm. and *Passiflora tonkinensis* W.J. de Wildeare the only species found in surrounding countries as well, and these primarily represent narrow range expansions beyond the borders of China south into Vietnam, or west into Myanmar or India.

The Chinese *Passiflora* are typically associated with limestone-rich soils and are most often found in wet, sunny openings within subtropical rainforest, along humid forest margins, or among large boulders on moist hillsides. These speciesgenerally require primary forests and are rarely found in secondary regrowth or disturbed habitat. The Asian *Passiflora* are found at elevations from 50 to 2000 meters but are most frequently associated with mid to upper elevations (1000–1500 meters). Population sizes are often quite small, with only a single plant observed over several kilometers (Krosnick 2006). This geographical isolation is compounded by the fact that the majority of *Passiflora* are self-incompatible (Ulmer and MacDougal 2004), which may effectively decrease population size even further. Their specialized habitat preferences and limited population size have undoubtedly contributed to the overall rarity of the Chinese *Passiflora*.

While not often discussed in the literature, a significant factor affecting the distribution of the native Chinese *Passiflora* has been deforestation that has occurred within the forests of China over the past 60 years. With the establishment of the People’s Republic of China in 1949, country-wide deforestation and forest degradation accelerated rapidly (Zaizhi 2001). This was due to intense logging for timber as well as fuelwood needs brought on by several important governmental initiatives (Zaizhi and Chokkalingam 2006). The first major degradation episode was from 1958–1961 during the Great Leap Forward and Iron-and-Steel Making campaigns, where communities set up large furnaces to make steel and used primary forest wood to make charcoal to feed these furnaces (Lang 2002, Zaizhi and Chokkalingam 2006). Between 1966 and 1976, the Great Cultural Revolution and governmental campaigns for self-sufficiency led to more deforestation for cultivation of corn and wheat, as well as additional fuelwood collection (Harkness 1988, Zaizhi and Chokkalingam 2006).

Because the Chinese species of *Passiflora* require primary forest and undisturbed habitats, deforestation and deterioration of forests throughout the subtropical southern provinces of Guangdong, Guangxi, Yunnan, Hainan, Jianxi, and Hunan would have been especially detrimental to these species. One species that appears to have been vulnerable to the effects of rapid deforestation is *Passiflora kwangtungensis* Merr. This species, originally described by Merrill in 1934, has since been documented in Guangdong (23 herbarium records), Guangxi (8 records), and Jiangxi Provinces (5 records). These are all provinces that experienced intense deforestation during the 1960’s and 1970’s. A total of just 35 specimens of *Passiflora kwangtungensis* were collected between 1924 and 1987, after which point all new collections ceased for this species. No additional collections of *Passiflora kwangtungensis* were obtained for 13 years, until a single specimen was observed and collected by Ye Huagu (*Ye 3381*, IBSC) in Guangdong Province in 2000. Even with this recent collection, when Krosnick and Deng performed fieldwork in 2003 visiting all recorded localities for *Passiflora kwangtungensis* in Guangdong Province, the forest habitats in each location had been cleared or heavily disturbed and the species was not located. At that time, it was assumed that this species was extremely rare, nearing extirpation in Guangdong Province and possibly near extinction throughout its entire range. Fortunately, in 2007, *Passiflora kwangtungensis* was reported by Yu in Hunan Province, a province where *Passiflora kwangtungensis* was not previously known to occur. Between 2007 and 2010, Yu observed approximately 14 plants in total across four localities in Hunan Province. His later collection of a single plant in 2010 (*Yu & Tan s.n.*, MO) represents a new locality record for *Passiflora kwangtungensis*, and quite possibly documents one of the last extant individuals of this species.

The recent high quality herbarium collection and photographs of fresh material that Yu made of *Passiflora kwangtungensis*, used in conjunction with herbarium material collected over the last 80 years, allow for the revision of Merrill’s original description to more accurately reflect this species with regard to morphology, ecology, and geographical distribution. Fresh DNA material collected from this specimen provides a new opportunity to examine the phylogenetic position of *Passiflora kwangtungensis* within supersection *Disemma* using ITS sequence data. In addition, conservation status assessments and recommendations are made for *Passiflora kwangtungensis* based on current distribution information according to ICBN criteria.

## Materials and methods

### Field observations

In 2004, botanical field work in Guangdong Province was completed by Krosnick and Deng. All known localities for *Passiflora kwangtungensis* in Guangdong were visited based on available herbarium specimen information at the time. Between the years of 2007–2010, Yu and accompanying students conducted field studies in the Nanling Mountains spanning four counties in south Hunan Province: Rucheng (Jiulongjiang National Forestry Park), Shuangpai (Wuxinling Forest Farm), Jingzhou (county nature reserve), and Jiangyong (provincial nature reserve), where they observed ca. 14 individual plants of *Passiflora kwangtungensis*. The greatest number of plants were observed at Rucheng (10 individuals), with just one or two individual plants seen at the Shuangpai, Jingzhou, and Jiangyong locations. Due to the rarity of the species, photos of *Passiflora kwangtungensis* were taken in lieu of herbarium specimens. A single herbarium specimen was collected in May 2010 from Jiulongjiang National Forestry Park (*Yu & Tan s.n.*, MO), as a voucher for morphological study and to provide tissue for DNA analysis.

### Morphological description

Krosnick (2006) examined 29 herbarium specimens representing material from the major herbaria with strengths in China to create a species description for *Passiflora kwangtungensis*. Eight additional specimens from IBSC, IBK, and LBG were examined by Deng and Krosnick for the current study, including the 2010 collection of *Yu & Tan s.n.* (MO). Thus, a total of 37 herbarium specimens from the following herbaria were examined: A, IBK, IBSC, KUN, L, LBG, MO, NY, PE, US. Extensive color photographs accompanying the *Yu & Tan s.n.* (MO) specimen were used to assist with color details in the species description.

### Geographical distribution

As none of the herbarium specimens examined contained primary GPS coordinates, an updated species distribution map was generated by inferring latitude and longitude coordinates using GOOGLE EARTH (Google 2012) or GeoNames Search (National Geospatial-Intelligence Agency 2012). Coordinates were inferred only where locality data was sufficiently detailed at the level of city, town, or village; thus, only 30 of the 37 herbarium specimens were used for the distribution map. Three additional points were added from populations observed directly by Yu during 2007–2010. Appendix 1 includes all herbarium specimen information with inferred latitude and longitude coordinates.

### Taxon sampling and outgroup selection

The monophyly of supersection *Disemma* was established using molecular data by Krosnick and Freudenstein (2005) and Krosnick (2006), with three monophyletic sections: *Disemma*, *Octandranthus*, and *Hollrungiella*. However, in those earlier analyses, *Passiflora kwangtungensis* was not included because fresh material was not available. Krosnick (2006) hypothesized that *Passiflora kwangtungensis*, once sampled,would fall within section *Octandranthus* based on morphology and geographical distribution. In the current analysis, supersection *Disemma* was fully represented with all 21 species currently recognized. Representative species from the following supersections in subgenus *Decaloba* were designated as outgroup taxa in this analysis: *Pterosperma* (L.E. Gilbert & J.M. MacDougal) J.M. MacDougal & Feuillet (1 sp.), *Multiflora* (Small) J.M. MacDougal & Feuillet (3 sp.), *Hahniopathanthus* (Harms) J.M. MacDougal & Feuillet (2 sp.), *Cieca* (Medik.) J.M. MacDougal & Feuillet (2 sp.), *Auriculata* J.M. MacDougal & Feuillet (1 sp.), *Bryonioides* (Harms) J.M. MacDougal & Feuillet (2 sp.),and *Decaloba* (DC.) J.M. MacDougal & Feuillet (8 sp.).Supersection *Pterosperma* (*Passiflora lancetillensis* J.M. MacDougal & Meerman) was designated as sister to the remaining taxa within subgenus *Decaloba* based on the position of this clade in previous analyses (Hansen et al. 2006, Yockteng and Nadot 2004). In total, the phylogenetic analysis included 40 species in subgenus *Decaloba*, with greatest sampling focused in supersection *Disemma*.

### DNA extraction, amplification and sequencing

Total genomic DNA was isolated from fresh leaf material or tissue preserved in silica gel and extracted using the CTAB method (Doyle and Doyle 1987) performed in microcentrifuge tubes, or with the DNeasy Plant Mini kit (Qiagen Inc., Valencia, CA). When necessary, DNA samples were further purified using the Elu-Quik DNA Purification Kit (Whatman Inc.,Piscataway, NJ), or the QIAquick PCR Purification Kit (Qiagen Inc., Valencia, CA). The nuclear ribosomal internal transcribed spacer region (nrITS) including ITS1, the 5.8S gene, and ITS2, was directly amplified using primers 5 and 4 of White et al. (1990). PCR reaction protocols for ITS followed Krosnick and Freudenstein (2005). Amplifications were purified by precipitating with 50 µl of 20% polyethylene glycol-2.5 M NaCl followed by two ethanol precipitations or by using Qiagen PCR Purification Kits (Qiagen Inc., Valencia, CA). Dideoxy cycle sequencing reactions were performed using BigDye Terminator version 3.1 chemistry (Applied Biosystems, Foster City, CA) scaled down to quarter reaction volume. Sequencing reactions were analyzed on an Applied Biosystems 3100 automated sequencer at The Ohio State University (Columbus, OH), or at Rancho Santa Ana Botanic Garden (Claremont, CA). Bidirectional sequence contigs were assembled and edited using GENEIOUS Pro v. 5.0.3 (Drummond et al. 2011), or by using SEQUENCHER v. 4.1.1 (Gene Codes Corporation 2000). All sequences were initially aligned using CLUSTAL W (Thompson et al. 1994), and manually adjusted using SE-AL (Rambout 2000). See Appendix 2 for complete list of taxa sampled, voucher information, and Genbank accession numbers. Appendix 3 contains the fully aligned dataset for all taxa as a NEXUS matrix file.

### Phylogenetic analyses

Unweighted Maximum Parsimony (MP) analyses were performed using WINCLADA (Beta) ver. 0.9.9 (Nixon 1999). All characters were treated as non-additive. Heuristic searches were performed using NONA ver. 2 (Goloboff 1999) with the following parameters: 10,000 trees held in memory (hold 10000), 5,000 tree bisection reconnection (TBR) replications (mult*5000), and using two starting trees per replication (hold/2). Resultant trees were summarized with a strict consensus. Branch support for the ITS analysis was assessed using 5,000 jackknife replicates in WINCLADA, with random character removal set at 37%. The heuristic searches for jackknife analyses utilized two TBR searches per replication (mult*2), using two starting trees per replicate (hold/2). Only clades with a frequency of 50% or higher were retained in the jackknife consensus. Jackknife support values were mapped directly onto the strict consensus for clades retained in both the jackknife and strict consensus topologies.

### Conservation status

Conservation recommendations were made following the ICBN guidelines for application of Red List categories and criteria (IUCN Standards and Petitions Subcommittee 2010).

## Results

### Phylogenetic analysis

**Figure 1.**
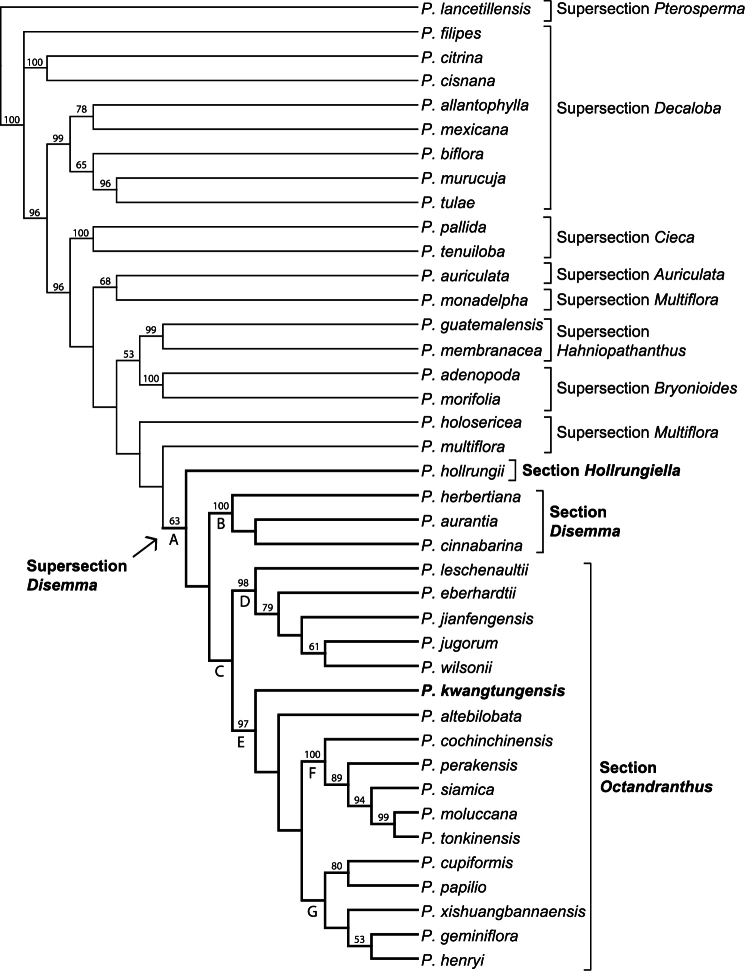
Strict consensus of two most parsimonious trees using ITS sequence data. Jackknife support above 50% listed above branches.

The aligned ITS dataset consisted of 801 characters, of which 272 were parsimony informative. The heuristic searches resulted in two most parsimonious trees (L=1,070 steps, CI=0.54, RI=0.71). One branch collapsed in the strict consensus of the two MP trees (Fig. 1). Results of the phylogenetic analysis support the monophyly of supersection *Disemma*, though with low jackknife support (63%; Fig. 1, clade A). Monophyly was strongly supported for supersections *Cieca* (100%), *Hahniopathanthus* (99%), and *Bryonioides* (100%). Supersection *Decaloba* is resolved as polyphyletic in this analysis, with one strongly supported (99%) clade containing *Passiflora allantophylla* Mast., *Passiflora mexicana* Juss., *Passiflora biflora* Lam., *Passiflora murucuja* L. and *Passiflora tulae* Urb.,a second clade consisting of *Passiflora citrina* J.M. MacDougaland *Passiflora cisnana* Harms (100%), and a single unresolved *Passiflora filipes* Benth. Supersection *Multiflora* is also polyphyletic, with *Passiflora holosericea* L. resolved as sister to *Passiflora multiflora* L.+ supersection *Disemma* (<50%), and then *Passiflora monadelpha* P. Jørg. & Holm-Niels. as sister to *Passiflora auriculata* Kunth (68%). Within supersection *Disemma*, *Passiflora hollrungii* K. Schum. is resolved as sister to the rest of the clade, which consists of two lineages, section *Disemma* (100%; Fig. 1, clade B), and section *Octandranthus* (<50%; Fig. 1, clade C). Within *Octandranthus*, two lineages are well supported: a clade of five species (98%; Fig. 1, clade D), and a second clade with the remaining 12 species (97%; Fig. 1, clade E). Although *Passiflora kwangtungensis* is resolved as sister to the remaining species in clade E, jackknife support for the position of *Passiflora kwangtungensis* and *Passiflora altebilobata* Hemsl. relative to remaining species is <50%. To further explore the placement of *Passiflora kwangtungensis* as sister to the remainder of clade E, another heuristic search using the same parameters was performed with *Passiflora altebilobata* removed from the dataset (data not shown). In that analysis, *Passiflora kwangtungensis* was still resolved as basal within clade E, suggesting that while jackknife support is low for its placement, the position of *Passiflora kwangtungensis* was not affected by the presence of *Passiflora altebilobata*. Within the remaining 10 species, two subclades appear: a Southeast Asian clade (100%; Fig. 1, clade F),and a Chinese clade (<50%; Fig. 1, clade G).

### Revised species description

**Figure 2.**
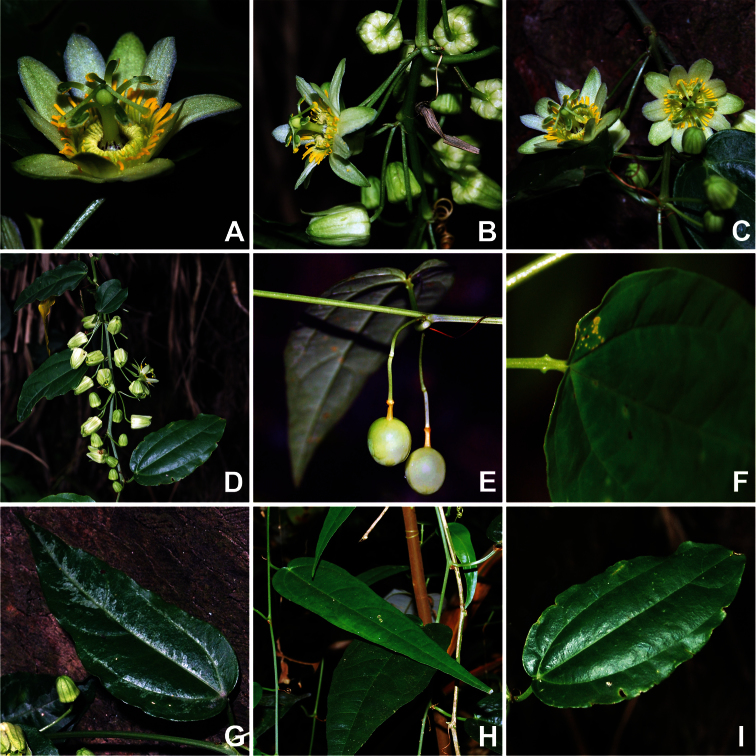
Floral and vegetative features of *Passiflora kwangtungensis*. **A** flower at anthesis **B** side view of open flower within inflorescence, three axillary inflorescences shown **C** arrangement of two flowers within axillary inflorescence **D** congested arrangement of individual axillary inflorescences, each with two to six flowers per inflorescence **E** immature fruit **F** papillate petiolar nectaries **G** mottled variegation along major veins in young leaf **H** lanceolate leaf shape associated with young growth **I** ovate leaf shape observed on older tissues. Photo credits: Xun-Lin Yu.

Based on the high quality photographs of living material (Fig. 2) and the additional herbarium specimens incorporated in the present study, a morphological description that more accurately reflects *Passiflora kwangtungensis* is presented here. Of particular note are color details that were not visible in the older herbarium specimens. Merrill (1934) suggested that the flowers of *Passiflora kwangtungensis* were white throughout, but it is now evident that flowers in this species have greenish-yellow sepals, whitish petals, an outer corona that is bright yellow in the upper half and yellow-green in the lower half, a yellow-green inner corona, and distinct brown flecks along the androgynophore and limen (Fig. 2A). Merrill also described the flowers as solitary in the axils of the leaves, but additional examination of herbarium material has revealed highly branched cymose inflorescences with up to 6 flowers per inflorescence. The inflorescences, when observed as they are naturally held on the plant (Fig. 2B–D), show a unique arrangement of third order branches on either side of the tendril (where the peduncle and terminal tendril are designated as first order, sensu Krosnick and Freudenstein 2005). Floral pedicels are of equal length and the terminal second order bud is sometimes absent. Merrill’s original description made no mention of fruits in *Passiflora kwangtungensis*. Although fresh samples were observed while immature, fruits in this species (Fig. 2E) appear similar in shape to other species in section *Octandranthus* in being relatively small (ca. 1 cm in diameter), globose, and paired.

The observation of fresh material has provided additional insights into *Passiflora kwangtungensis* with regard to vegetative characters.The petiole has two paired papillate nectaries near the apex of the petiole (Fig. 2F). While the original description did note that the leaves are 3-nerved (Fig. 2F–G), the fresh material reveals a unique mottled variegation along the veins in younger leaves. Two distinct leaf shapes, lanceolate and ovate, are observed in the fresh material (Fig. 2G–I). The more lanceolate shape is associated with the juvenile growth form, while the ovate shape is observed on older portions of the plant.

As part of the revised species description presented below, information on phenology, ecology, and geographical distribution is presented to facilitate identification and conservation of this species in the field.

#### Passiflora kwangtungensis

Merr., Lingnan Sc. Journ. 13: 38. 1934

http://species-id.net/wiki/Passiflora_kwangtungensis

Figures 1 and 2

##### Type.

CHINA. Guangdong: Tsungfa-Lungmoon Districts, Sam Kok Shan, Ka Wong Kwa, 29 May 1932, *Tsang 20609* (holotype: NY! [NY-110492], isotype: NY! [NY-110491], PE! [PE-25522]).

##### Description.

Slender climber, glabrous throughout; stems terete. Stipules 1.0 × 0.5 mm, setaceous; petioles 1.0–2.0 cm long, biglandular in the upper half, the nectaries 0.3–1.0 mm in diam., papillate; laminas 9.0–13.0 cm × 2.0–5.0 cm, lanceolate to ovate, cordate at the base, apex acute to acuminate, midvein with a 1 mm mucro, margins entire, diffuse white variegation sometimes present along major veins; laminar nectaries 0.2–0.5 mm in diam, (0–) 2–7, scattered submarginally on abaxial surface. Tendrils well developed in mature shoots, green; inflorescences cymose, branched through the third order, (1–) 4–6 flowered; peduncle absent, pedicels 1.3–2.5 cm long, with an articulation 1.0–2.0 cm from the base; inflorescence bracts 1.0 mm × 0.5 mm, linear. Flower buds ovoid, the largest buds 5.0 mm × 3.0 mm; flowers erect; hypanthium 5.0 mm in diam.; sepals 5, 5.0–7.0 mm × 2.5–3.0 mm, lanceolate, glabrous, greenish-yellow, apex acute; petals 5, 4.0–6.0 mm × 2.0 mm, narrowly oblong-lanceolate, greenish-white, apex acute; coronal filaments in two series, outer series 3.0–5.0 mm long, filiform, yellow-green in lower half, yellow in upper half, inner series 1.0–2.0 mm long, filiform, clavate at apex, yellow-green throughout; operculum 1.0–2.0 mm tall, membranous, plicate, incurved towards the androgynophore, yellow-green, the inner margin fimbriate; limen 3.0 mm in diam., outer perimeter with 1 mm tall rim; nectar ring 1.0–2.0 mm wide; stamens 5, staminal filaments connate 4.0 mm along androgynophore, the free portions 4.0 mm long, green, the base flecked with brown spots 0.5–1.0 mm long; anthers 2.0 mm × 1.0 mm, green; ovary 3.0 mm × 1.5 mm, ovoid, sessile on the androgynophore, glabrous, green; styles 3, 3.0 mm long excluding stigmas; stigmas ovoid, 0.5 mm in diam. Fruit 1.0 cm in diam., globose, blue at maturity; arils unknown. Seeds unknown.

##### Phenology.

Flowering May; fruiting May–June.

##### Distribution

**Figure 3.**
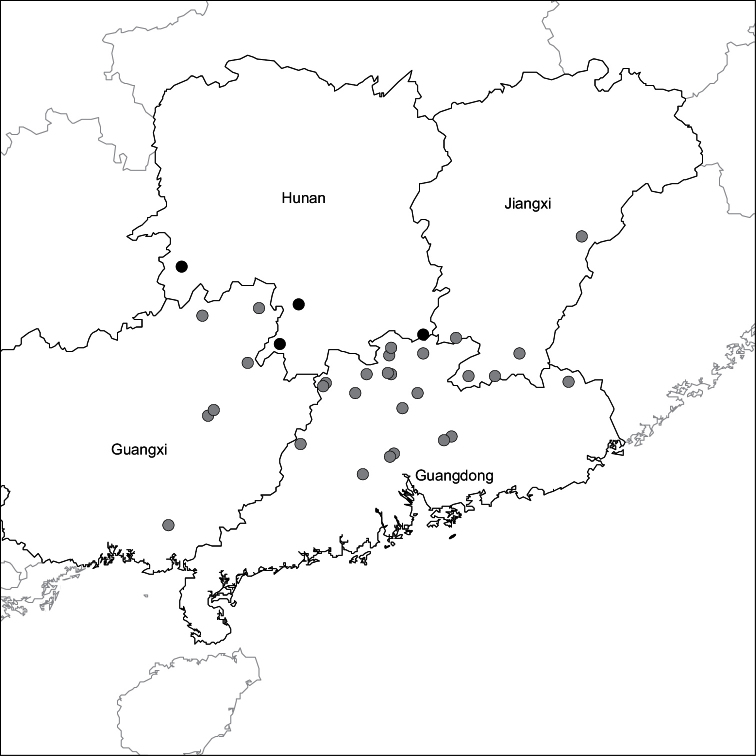
Distribution of *Passiflora kwangtungensis* in China. Grey circles indicate localities taken from herbarium specimens. Black circles indicate populations observed by Yu from 2007–2010.

(Figure 3). Endemic to China in Guangdong, Guangxi, Jiangxi and Hunan Provinces; rare.

##### Ecology.

*Passiflora kwangtungensis* is observed most frequently on hillsides in thickets, along roadsides in forest valleys, or along primary forest margins. This species prefers wet, sandy soils, and is scandent along the ground, sometimes climbing onto low shrubs or tree trunks. Elevation ranges from 500–1000 m.

##### Vernacular names.

China:“Guang dong xi fan lian” (Wang et al. 2007).

##### Specimens examined.

**CHINA. Guangdong**: 800 m, Anon. 915 (PE); Yangshan Xian, Wuyuan Xiang, Tianjingshan, *Deng 1370* (IBSC); Nanxiong Xian, Baishun Xiang, Danankeng, 300–400 m, *Deng 6213* (IBSC); Yingde Xian, Shakou Xiang, Huashuishan, 580 m, *Liang 84483* (IBSC, PE); Lechang, Jiufeng, Lianan, *Lo 1084* (IBSC); Longmen Xian, Nankunshan, Zhongping, Zhukeng, Shihuixiezi, *Lo 1782* (IBSC); Lian Shan Town, *Nang 659* (IBK, IBSC); Yangshan, *Nanling Expedition 1349* (IBSC); Longmen, 350 m, Nanling Expedition 2006 (IBSC); Jiaoling, *Nanling Expedition 2273* (IBSC); Yangshan Xian, Chengjia, *Tam & Huang 359* (IBSC); Lianshan Xian, Hedong Xiang, Huangniushan, 880 m, *Tam 58338* (IBSC); K’I Ravine, *To & Ts’ang 12274* (A); Lung T’an Xian, *To & Ts’ang 2035372* (L); Lung T’au Mtn., *To Kang et al. 275* (US); Lung T’an Mtn., *To Kang et al. 535* (US); Tsungfa-Lungmoon Districts, Sam Kok Shan, Ka Wong Kwa, *Tso 20749* (NY [paratype]). Lianshan Xian, Shangshuai Zhen, Jinjiling, *Ye 3381* (IBSC); Ruyuan, Daqiao, *Yue-71 Expedition 355* (IBSC); Ruyuan Xian, Ruyang, Baimakeng, 1200 m, *Yue-73 Expedition 720* (IBSC); Fengkai Xian, Qixing, *Yue-74 Expedition 4958* (IBSC); Heping, *Zhang 705* (IBSC); **Guangxi**: Jinxiu Xian, 1000 m, *Dayaoshan Expedition 12445* (IBK); Jinxiu Xian, 500 m, *Dayaoshan Expedition 811616* (IBK); Gongcheng, *Gongcheng Expedition 0179* (IBK); Longsheng Xian, *Qin & Li 70609* (IBK, IBSC); Ku Chun, Yao Shan (Dayao Shan Mtns.), *Sin 21066* (IBSC); Kuchen, *Sin 21283* (IBSC); Kuchen, *Sin 21407* (IBSC); Quanzhou, near Baiyunan, Tsang 27737 (IBSC, US); **Hunan**: Jiulongjiang National Forest Park, Rucheng Xian, 520 m, *Yu & Tan s.n.* (MO); **Jiangxi**: Anyuan Xian, Huangdi, *Lai 2273* (LBG); Quannan Xian, Zhushan Xiang, Yaoshan, Longwei, 800 m, *Lai 768* (LBG); Lichuan County, Hong Ling Qu Kongdau Xiang, *Nie et al.* *2773* (KUN); Dayu Xian, Yaofu, 650 m, *Yue 1297* (IBSC; KUN).

## Discussion

### Phylogenetic position of *Passiflora kwangtungensis*

Based on the strict consensus of the ITS data presented here (Fig. 1), both supersection *Disemma* (clade A) and section *Octandranthus* (clade C) are resolved as monophyletic, though with low jackknife support. Within section *Octandranthus*, *Passiflora kwangtungensis* is strongly supported as a member of clade E, which consists of species from India, Nepal, China, and Southeast Asia. *Passiflora kwangtungensis* and *Passiflora altebilobata* form a basal grade leading to a Southeast Asian clade (clade F) and a Chinese clade (clade G). However, jackknife support values are quite low for several key nodes within the ITS phylogeny presented here, suggesting that alternative topologies may be obtained as more loci are included. Therefore, while taxon sampling for supersection *Disemma* is complete with regard to the ITS dataset, it is not yet possible to make strong conclusions regarding relationships within *Disemma* or about the phylogenetic position of *Passiflora kwangtungensis* in section *Octandranthus*. The addition of nuclear and chloroplast sequence data for *Passiflora kwangtungensis* will allow for more thorough insights into the evolutionary position of this species within section *Octandranthus*.

Supersection *Disemma* is a difficult lineage to study from a morphological standpoint because there are no clear synapomorphies that distinguish these 21 species as a group from the rest of subgenus *Decaloba*. Moreover, there seems to be a high rate of character transformation in this lineage, such that even closely related species appear quite distinct with regard to key floral and vegetative features. For example, within clade D, *Passiflora eberhardtii* has the smallest flowers in the supersection (ca. 1 cm or less in diameter), large cordate leaves with scattered abaxial nectaries, and flattened petiolar nectaries. This species is sister to a clade containing *Passiflora jianfengensis* S.M. Hwang & Q. Huang, *Passiflora jugorum*, and *Passiflora wilsonii*, all of which have flowers 3 cm or greater in diameter, leaves that are more or less truncate, abaxial nectaries in pairs, and petioles with peg-shaped glands. Similarly, placement of *Passiflora kwangtungensis* within supersection *Disemma* is challenging because while this species displays characters that might be considered plesiomorphic for clade E, it also exhibits many morphological similarities (inflorescence structure, floral coloration, and petiolar nectary shape) to both *Passiflora henryi* Hemsl. and *Passiflora geminiflora* D. Don, both of which occupy relatively derived positions in clade G. Thus, it is useful to consider the similarities of *Passiflora kwangtungensis* to the remainder of clade E as a whole (*Passiflora altebilobata*+ clades F, G), as well the similarities of this species to *Passiflora henryi* and *Passiflora geminiflora*.

Considering first the placement of *Passiflora kwangtungensis* as basal within clade E, a number of features observed in *Passiflora kwangtungensis* could be viewed as plesiomorphic for this clade. *Passiflora kwangtungensis* has small flowers that are generally no larger than 2 cm in diameter. Seven of the 12 species in clade E have small flowers (less than 2.5 cm in diameter), while the five Southeast Asian species (clade F) have much larger flowers, generally 3–5 cm in diameter. Larger flower size could represent a synapomorphy for clade F, while for the rest of clade E flowers could have remained small. All species in clade E display inflorescence branching through at least the second order, and all species have at least two flowers per inflorescence. Branching in *Passiflora kwangtungensis* may be through the third order, with one to four flowers per inflorescence. However, there is great variation in the extent of branching across the species in clade E. For example, *Passiflora altebilobata* has branching through the fourth order and up to 11 flowers per inflorescence, while inflorescences in *Passiflora cupiformis* may have up to 18 flowers. Leaves in *Passiflora kwangtungensis* range from lanceolate to ovate, simple shapes that could easily be modified to create the various forms observed across the clade. *Passiflora altebilobata* (clade E) and *Passiflora xishuangbannaensis* Krosnick (clade G) are perhaps the most specialized with deeply bilobed leaves, but this shape could be readily achieved through truncation of the midvein if starting from an ovate leaf form. The leaves of *Passiflora kwangtungensis* have submarginal abaxial nectaries, a feature which is observed in all species across clade E. Should its basal position continue to be supported as additional loci are sequenced, the morphological features of *Passiflora kwangtungensis* described here would be consistent with character traits in the other 11 species in clade E, highlighting the notable morphological plasticity in supersection *Disemma*.

Alternatively, there are three morphological similarities shared among *Passiflora kwangtungensis*, *Passiflora henryi*, and *Passiflora geminiflora* that are suggestive of a close relationship between these species. First, *Passiflora kwangtungensis* (Fig. 2B–D), *Passiflora henryi*, and *Passiflora geminiflora* display many similarities with regard to their inflorescence architecture. They all have cymose inflorescences branched through the third or fourth order. Within the inflorescence, third order flowers sometimes appear to be arranged in pairs, caused when the second order bud is aborted. This condition is commonly observed in *Passiflora geminiflora* and somewhat less commonly in *Passiflora henryi*. Pedicels within the inflorescence are of more or less equal lengths and held at the widest angle possible from one another, which results in the inflorescences appearing as mirror images of one another on either side of the central tendril. This differs from other species in section *Octandranthus* that have fasciculate inflorescences caused by the presence of sequentially shorter pedicels as branching order increases. Second, *Passiflora kwangtungensis* (Fig. 2A), *Passiflora henryi*, and *Passiflora geminiflora* each exhibit narrow flecks of brown coloration ca. 1 mm in length along the androgynophore and limen surface. Third, *Passiflora kwangtungensis* has papillate to narrowly peg-shaped petiolar nectaries (Fig. 2F), which are also observed in *Passiflora henryi* and *Passiflora geminiflora*. Should additional data resolve *Passiflora kwangtungensis* with *Passiflora henryi* and *Passiflora geminiflora*, these similarities would represent synapomorphies for that clade. These features are strongly suggestive of an evolutionary connection among the three species, or at the very least, an interesting convergence of form.

### Geographical distribution

*Passiflora kwangtungensis* was originally described based on two herbarium specimens (*Tsang 20609* holotype, *Tso 20749* paratype) collected in Guangdong Province. Even in the original description, Merrill (1934) noted the affinities between *Passiflora geminiflora* (as syn. *Passiflora nepalensis* Walp.) with *Passiflora kwangtungensis*. In 1940, Chun, in “Flora of Kwangtung and South-Eastern China, III” noted a new collection of *Passiflora kwangtungensis* in Guangxi Province. Later, in 1972, De Wilde cited three additional specimens from Guangdong. In 1984, Bao cited two new records for Jiangxi and one for Guangxi. The geographical distribution of *Passiflora kwangtungensis* was cited as Guangdong, Guangxi, and Jiangxi by both Krosnick (2006) and Wang et al. (2007). The most recent specimens available (Guangdong: 2000, *Ye 3381*, IBSC; 2010, Hunan: *Yu & Tan s.n.*, MO) suggest that this species is still extant, though quite rare, and may be found in a narrow range along the border of Guangdong and Hunan Provinces.

Historically, *Passiflora kwangtungensis* appears to have been most abundant in Guangdong Province, with 23 of the 37 localities from this province. Given the high number of deforestation events that have occurred in southern China since 1958, it seems plausible that the decreasing numbers of collections each year for *Passiflora kwangtungensis* was correlated to the abundance of suitable habitat available in its native range. It is possible that these declining collections may simply reflect a decrease in botanical field work in Guangdong, Guangxi, and Jiangxi Provinces. However, given the gradual decline in numbers of *Passiflora kwangtungensis* specimens collected from the 1960’s through the 1980’s and the complete absence of collections after 1987, it seems more likely the result of reduced available habitat for an already rare, obligately out-crossing species being pushed to the brink of extinction throughout its range. The two most recent collections made in 2000 and 2010 are along the border of Guangdong and Hunan Province in the Nanling Mountain Range (Fig. 3). Based on the field observations of Yu during 2007–2012, it appears that the ca. 14 individual plants observed in Hunan Province may be some of the last remaining extant individuals of *Passiflora kwangtungensis*.

### Conservation

Under the IUCN Red List guidelines (IUCN Standards and Petitions Subcommittee 2010), *Passiflora kwangtungensis* should be classified as **CR C1+C2a(i); D**, or critically endangered, based on two assessment criteria, C and D. With respect to criterion C, small population size and decline: the number of mature individuals known for *Passiflora kwangtungensis* is less than 250 in total, with just 14 plants observed in Hunan over three years of surveys. Within category C, *Passiflora kwangtungensis* should be classified as C1, an estimated continuing population decline of at least 25% in 3 years or 1 generation, because this species is self-incompatible and exists in extremely fragmented environments which restrict gene flow. It is important to note the definition of population according to the IUCN (2010) is the total number of individuals in a taxon, rather than the number of individuals at a given location. In a traditional sense, population sizes of *Passiflora kwangtungensis* are even smaller (ca. 1–2 individuals in three observed populations, 10 maximum for the largest population according to Yu’s observations). Given these limitations, it is likely there will be a decline of the total population size over the next generation (which could be 5–10 years based on most *Passiflora* species). Within category C, *Passiflora kwangtungensis* can also be classified as C2, a continuing decline and a(i) number of mature individuals in each subpopulation less than 50. Given that only 36 herbarium collections have been made since 1924 and ca. 14 plants are currently known from Hunan, a realistic estimate of the total population size for *Passiflora kwangtungensis* would be 50 or fewer, optimistically.

Under criterion D, very small or restricted population: *Passiflora kwangtungensis* should be classified as D, number of mature individuals less than 50. This species, previously feared to be extinct throughout its native range, is surviving in isolated pockets along primary forest margins, or quality habitat on undamaged hillsides throughout the Nanling Mountains. There is likely very little gene flow among the subpopulations, and even if the species are self-compatible, genetic diversity would be assumed to be quite low due to inbreeding. Fortunately, three of the four locations where *Passiflora kwangtungensis* was observed are in county, provincial, or national park reserves. This gives them some protection from habitat destruction but cannot ensure their survival due to reproductive isolation caused by low population numbers.

While the highest Red List conservation status a species qualifies for should be used, *Passiflora kwangtungensis* would also qualify as endangered under criterion A2abc, where A2 specifies a ≥50% decline over the longer of 10 years or three generations, and where population reduction was observed or inferred to have occurred in the past and the causes of reduction may not have ceased, may not be reversible, and may not be understood. The “abc” is determined based on a, direct observation, b, an index of abundance appropriate to the taxon, and c, a decline in the extent of occurrence and habitat quality. If herbarium specimens are taken as evidence, a clear drop off in the number of collections made occurs from the late 1980s forward. Conservatively, the lower number of specimens collected is assumed to reflect reduced population numbers, as opposed to reduced collecting efforts by scientists in the region. As several important floristic works focused on China have emerged during the 1980’s and 1990’s (Chen 1987, Lin 1993, Wu 1984, Wu and Raven 1994), it seems the former explanation is more likely than the latter. Moreover, herbarium specimen records indicate a clear decline in the extent of occurrence in *Passiflora kwangtungensis* throughout its originally described range; much of this is likely due to decreased habitat availability brought on during the deforestation campaigns spanning the 1950s to 1970s. *Passiflora kwangtungensis* does not appear to have ever been truly abundant in its native habitat, at least given evidence from specimens collected from 1924 onward. It may be that population numbers of *Passiflora kwangtungensis* were reduced below a sustainable size during the deforestation efforts that occurred mid to late-century, thus setting off the observed decline in the late 1980s and onward. Taken together, the evidence suggests that this species has been extirpated, or is nearing extirpation, from Guangdong, Guangxi, and Jiangxi Provinces and is currently surviving only in isolated pockets of refugial habitat in Hunan.

In general, *Passiflora* grow quite well from stem cuttings. Both in situ and ex situ conservation methods would be recommended with immediate implementation to protect the remaining individuals of *Passiflora kwangtungensis* from what seems to be near-certain extinction. Seeds, if produced, should be germinated and maintained in cultivation at local botanical gardens (such as IBSC) where soil type and other ecological factors will be most favorable for their survival. Further exploration is needed in Hunan, Jiangxi, and Guangxi Provinces to see if additional refugial populations still exist; if so, particular effort should be placed on cultivation of stem cuttings and eventual cross-pollination with the Hunan material to increase the genetic diversity of the material in cultivation. The case of *Passiflora kwangtungensis* represents a rare opportunity where botanists have the chance to assist in bringing a plant back from the brink of extinction. We hope that the information presented here will facilitate the protection and conservation of this species. This manuscript will also be presented as part of the application for placement of *Passiflora kwangtungensis* as critically endangered on the IUCN Red List, a recognition that will confer additional protection and increased awareness regarding the status of this species.

## Supplementary Material

XML Treatment for Passiflora kwangtungensis

## Appendix 1

Herbarium specimen localities with inferred geographical coordinates. (doi: 10.3897/phytokeys.23.3497.app1) File format: Microsoft Excel document (xls).

**Explanation note:** Localities used for mapping the geographical distribution of *Passiflora kwangtungensis*.

## Appendix 2

List of species of *Passiflora* subgenus *Decaloba* used in the molecular phylogenetic analysis of ITS. (doi: 10.3897/phytokeys.23.3497.app2) File format: Microsoft Word document (docx).

**Explanation note:** Information includes supersection, section, voucher information (collector, collection number, and herbarium acronym), and GenBank accession numbers for ITS sequences.

## Appendix 3

Aligned ITS dataset used in phylogenetic analysis of *Passiflora kwangtungensis*. (doi: 10.3897/phytokeys.23.3497.app3) File format: Nexus file (nex).
