# Supplementary material for: The rediscovery of Passiflora kwangtungensis Merr. (subgenus Decaloba supersection Disemma): a critically endangered Chinese endemic
Source: PhytoKeys. 2013 Jun 12;(23):55–74. doi: 10.3897/phytokeys.23.3497 (PMC3690916; doi:10.3897/phytokeys.23.3497)
Supplement: Supplementary file 3 — List of species of Passiflora subgenus Decaloba used in the molecular phylogenetic analysis of ITS. (doi: 10.3897/phytokeys.23.3497.app2) File format: Microsoft Word document (docx). [file PhytoKeys-023-055-s002.docx]

Appendix 2. List of species of *Passiflora* subgenus *Decaloba* sequenced for ITS including supersection, section, voucher information including collector, collection number, and herbarium acryoynm, and GenBank accession numbers for ITS sequences.

| **Supersection** | **Section** | **Species** | **Voucher** | **GenBank**  **ITS accession reference** |
| --- | --- | --- | --- | --- |
| *Passiflora* supersect. *Auriculata* J. M. MacDougal & Feuillet |  | *Passiflora auriculata* Kunth | *Krosnick 350* (OS) | DQ284532 |
| *Passiflora* supersect. *Bryonioides* (Harms) J. M. MacDougal & Feuillet |  | *Passiflora morifolia* Mast*.* | *Krosnick 311* (OS) | DQ284535 |
|  |  | *Passiflora adenopoda* DC. | *Krosnick 258* (OS) | AY632702 |
| *Passiflora* supersect. *Cieca* (Medic.) J. M. MacDougal & Feuillet |  | *Passiflora pallida* L. | *Krosnick 18* (OS) | DQ458084 |
|  |  | *Passiflora tenuiloba* Engelm. | *Goldman 1770* (BH) | AY632719 |
| *Passiflora* supersect. *Decaloba* (DC.) J. M. MacDougal & Feuillet | *Passiflora* sect. *Decaloba* DC. | *Passiflora allantophylla* Mast. | *Krosnick 25* (OS) | DQ458069 |
|  |  | *Passiflora biflora* Lam. | *Krosnick 260* (OS) | AY632705 |
|  |  | *Passiflora filipes* Benth. | *Goldman 2153* (BH) | AY632709 |
|  |  | *Passiflora mexicana* A. Juss. | *Goldman 1774* (BH) | AY632713 |
|  |  | *Passiflora murucuja* L. | *Kay 217* (MO) | AY648559 |
|  |  | *Passiflora tulae* Urb. | Muschner et al. (2003) | AY102352, AY102372 |
|  | *Passiflora* sect. *Xerogona* (Raf.) Killip | *Passiflora citrina* J. M. MacDougal | *Krosnick 23* (OS) | DQ458083 |
|  |  | *Passiflora cisnana* Harms | *Krosnick 27* (OS) | AY632716 |
| *Passiflora* supersect. *Disemma* (Labill.) J. M. MacDougal & Feuillet | *Passiflora* sect. *Disemma* (Labill.) J. M. MacDougal & Feuillet | *Passiflora aurantia* G. Forst. | *Krosnick 24* (OS) | AY632704 |
|  |  | *Passiflora cinnabarina* Lindl. | *Butler 66949* (CBG) | AY632706 |
|  |  | *Passiflora herbertiana* Ker Gawl. | *Krosnick 255* (OS) | AY632711 |
|  | *Passiflora* sect. *Hollrungiella* Harms | *Passiflora hollrungii* K. Schum. | *Banka and Krosnick 2051* (LAE) | DQ458081 |
|  | *Passiflora* sect. *Octandranthus* Harms | *Passiflora altebilobata* Hemsl. | *Krosnick 03* (OS) | DQ458078 |
|  |  | *Passiflora cochinchinensis* Spreng*.* | *Krosnick 198* (OS) | AY632714 |
|  |  | *Passiflora cupiformis* Mast*.* | *Krosnick 253* (OS) | AY632708 |
|  |  | *Passiflora eberhardtii* Gagnep*.* | *Krosnick 16* (OS) | DQ458073 |
|  |  | *Passiflora geminiflora* DC. | *Krosnick 337* (OS) | DQ458075 |
|  |  | *Passiflora henryi* Hemsl. | *Krosnick 08* (OS) | AY632710 |
|  |  | *Passiflora jianfengensis* S. M. Hwang & Q. Huang | *Krosnick 293* (OS) | DQ458077 |
|  |  | *Passiflora jugorum* W. W. Sm. | *Krosnick 15* (OS) | AY632712 |
|  |  | *Passiflora kwangtungensis* Merr. | *Yu & Tan, s.n.* (MO) | KF207865 |
|  |  | *Passiflora leschenaultii* DC*.* | *Krosnick 342* (OS) | DQ458079 |
|  |  | *Passiflora moluccana var. glaberrima* (Gagnep.) De Wilde | *Krosnick 13* (OS) | DQ284536 |
|  |  | *Passiflora papilio* Li | *Krosnick 252* (OS) | DQ458074 |
|  |  | *Passiflora perakensis* Hallier f. | *Krosnick 314* (OS) | DQ087422 |
|  |  | *Passiflora siamica* Craib | *Krosnick 320* (OS) | DQ458212 |
|  |  | *Passiflora tonkinensis* De Wilde | *Krosnick 348* (OS) | DQ087424 |
|  |  | *Passiflora wilsonii* Hemsl. | *Wen 5973* (F) | DQ087425 |
|  |  | *Passiflora xishuangbannaensis* Krosnick | *Krosnick 254* (OS) | DQ458071 |
| *Passiflora* supersect. *Hahniopathanthus* (Harms) J. M. MacDougal & Feuillet |  | *Passiflora guatemalensis* S. Watson | *Krosnick 347* (OS) | DQ087419 |
|  |  | *Passiflora membranacea* Benth*.* | *Krosnick 19* (OS) | AY632701 |
| *Passiflora* supersect. *Multiflora* (Small) J. M. MacDougal & Feuillet |  | *Passiflora holosericea* L. | *Krosnick 328* (OS) | DQ087417 |
|  |  | *Passiflora monadelpha* P. M. Jorgensen & L. B. Holm-Nielsen | *Jorgensen, Ulloa, Narvaez & Lara 1774* (MO) | DQ087418 |
|  |  | *Passiflora multiflora* L. | *Goldman 2164* (BH) | AY632715 |
| *Passiflora* supersect. *Pterosperma* L. E. Gilbert & J.M. MacDougal |  | *Passiflora lancetillensis* J. M. MacDougal & Meerman | *Meerman s.n.* (MO) | DQ458065 |
